# Wearable Potentiometric Sensors for Medical Applications

**DOI:** 10.3390/s19020363

**Published:** 2019-01-17

**Authors:** María Cuartero, Marc Parrilla, Gaston A. Crespo

**Affiliations:** Department of Chemistry, KTH Royal Institute of Technology, Teknikringen 30, SE-10044 Stockholm, Sweden; marcpp@kth.se

**Keywords:** potentiometry, all-solid-state, wearable sensing, sampling cell, iontophoresis, sweat analysis, clinical decision-making, cystic fibrosis

## Abstract

Wearable potentiometric sensors have received considerable attention owing to their great potential in a wide range of physiological and clinical applications, particularly involving ion detection in sweat. Despite the significant progress in the manner that potentiometric sensors are integrated in wearable devices, in terms of materials and fabrication approaches, there is yet plenty of room for improvement in the strategy adopted for the sample collection. Essentially, this involves a fluidic sampling cell for continuous sweat analysis during sport performance or sweat accumulation via iontophoresis induction for one-spot measurements in medical settings. Even though the majority of the reported papers from the last five years describe on-body tests of wearable potentiometric sensors while the individual is practicing a physical activity, the medical utilization of these devices has been demonstrated on very few occasions and only in the context of cystic fibrosis diagnosis. In this sense, it may be important to explore the implementation of wearable potentiometric sensors into the analysis of other biofluids, such as saliva, tears and urine, as herein discussed. While the fabrication and uses of wearable potentiometric sensors vary widely, there are many common issues related to the analytical characterization of such devices that must be consciously addressed, especially in terms of sensor calibration and the validation of on-body measurements. After the assessment of key wearable potentiometric sensors reported over the last five years, with particular attention paid to those for medical applications, the present review offers tentative guidance regarding the characterization of analytical performance as well as analytical and clinical validations, thereby aiming at generating debate in the scientific community to allow for the establishment of well-conceived protocols.

## 1. Introduction

Potentiometry based on ion-selective electrodes (ISEs) is a well-established analytical technique present in almost every laboratory. The wide use of ISEs has its roots in the superior analytical features that permit the highly and versatile detection of ions on the benchtop within a broad dynamic range of response (3–5 orders of magnitude) and rapid analysis time, which is also accompanied by remarkable affordability in terms of simplicity and low cost [1,2]. Furthermore, the acquired signal at close to zero-current conditions, i.e., electromotive force (EMF) *versus* time, offers an easy interpretation by non-expert end-users who are able to analyze ion content in real (and synthetic) samples in a very straightforward manner [1].

**Figure 1:**
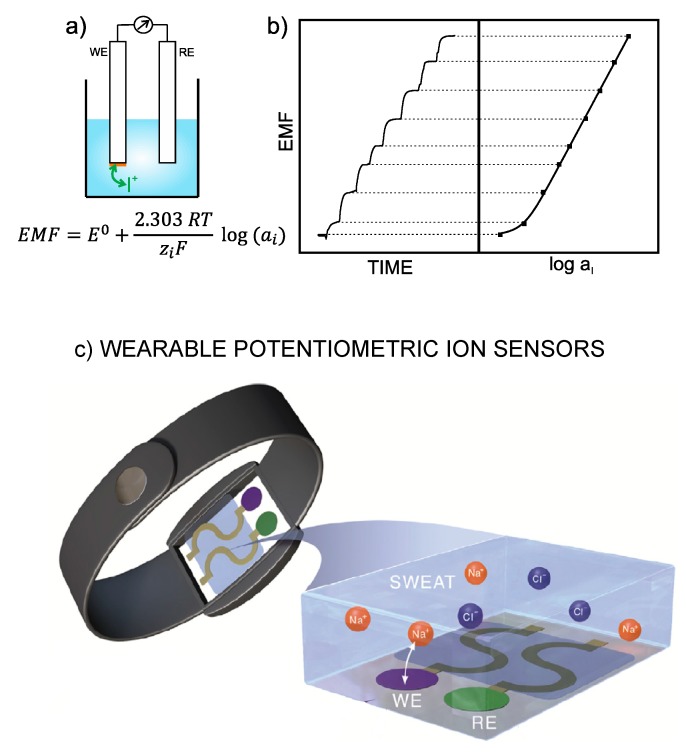
(**a**) Illustration of a general electrochemical cell based on a potentiometric electrode (also called working electrode, WE, or indicator electrode), which comprises an ion-selective membrane (sketched in orange), and the reference electrode (RE). Whereas the potential provided by the RE is constant, the potential displayed by the WE depends on the concentration of the ion analyte (the cation, I^+^, is shown in this case) in the bulk solution. EMF=electromotive force, ISE=ion-selective electrode. (**b**) Typical trace of a dynamic potential response of an ISE for increasing concentrations of the ion analyte (a cation in this case) together with the corresponding calibration graph (logarithmic activity *versus* potential). Inset equation: Nernst equation, where E^0^ is the standard potential of the cell, R is the gas constant, T is the temperature, z_i_ is the charge of the ion analyte and F is the Faraday constant. For the calibration curve, E^0^ corresponds to the intercept and $\frac{2.303\;RT}{z_{i}F}$ to the slope. (**c**) Illustration of a wearable potentiometric ion sensor based on a bracelet that is modified with the WE and RE, which are in contact with the sweat where the exchange of the target ion (in this case ${\mathrm{Na}}^{+}$) with the selective membrane in the WE is responsible for the potentiometric response. Reprinted from [4], Copyright 2019, with permission from Elsevier.

With ISEs, the analytical information is obtained based on the translation of an ion-exchange event into a voltage signal [1]. Thus, the electrode is designed in such a way as to recognize any perturbation (of a sufficient magnitude) occurring in the local equilibrium established at the interface between an ion-selective membrane (ISM) and the sample solution. Consequently, a change in the activity of the primary ion in the bulk sample solution produces variation in membrane potential. The difference between this potential and that provided by the reference electrode leads to the potentiometric readout, i.e., EMF of the electrochemical cell (Figure 1a). Besides this, the concentration dependence of the potentiometric signal with the activity of the primary ion is described by the Nernst equation, and the plot of the EMF *versus* logarithmic activity provides *per se* the calibration graph of the ISE (Figure 1b) [3], which is employed to detect unknown ion concentrations in (aqueous) samples.

Beyond the well-entrenched pH and fluoride ISEs, which consist of a silicate glass membrane and LaF_3_ crystalline membrane doped with europium, respectively, ISEs based on plasticized polymeric membranes (termed ISMs) are the most widely used today. In addition, the all-solid-state configuration of the electrode is preferred over the setup comprising an inner-filling solution because, while maintaining the same analytical features, the all-solid-state concept offers a series of unique advantages [1,5,6]: (i) The size of ISEs is scaled down to the nano-level [2]; (ii) Easy implementation in fluidic systems, which fosters in-line incorporation of special pre-treatments [7,8]; (iii) Requirement of very low sample volumes (just a few microliters) [2]; (iv) Extremely wide catalogue of shapes and configurations (including flat electrodes) [6]; (v) Compatibility with many electrode substrates permits integration into very distinct platforms (even objects) already developed, such as paper, ceramics, plastic, rubbers and textiles, among others [6,9,10,11].

Within this context, while fundamental studies on potentiometric ISEs were important issues during the initial decades of their maturation (Pungor and Simon-Pretsch schools and subsequent disciples, 1970–1990) [1,2,12], we are now immersed in the era for the exploitation of these fundamental concepts at the applied level. As the all-solid-state configuration is the ‘holy grail’ towards this purpose, the main goal is now likely established on the basis of implementing potentiometric ISEs in every single scenario with a clear need for ion analyses. For instance, this is the case of *in situ* water sensing [13] and wearable on-body devices [4]. The key point relies on generating useful chemical information in real time, and, hence, it is crucial to achieve total decentralization of acquisition and interpretation of the analytical signal [12,14].

Focusing now on wearable potentiometric sensors, the undeniable need for trustable data in relation to the body status of an individual during sport activity or in patients’ health checks has prompted a rapid rise in the reported papers in this field over the last ten years [4]. Interestingly, all-solid-state potentiometry is the only technology that can be currently integrated into a portable device, i.e., a fully decentralized system, for on-body analysis of ions in different biological fluids. Indeed, annual reports detailing the wearable chemical sensors market point out the potential of electrochemical sensors, in general, and also with potentiometric readout in particular, thereby striving towards the development of a new era of profitable devices. Furthermore, they are predicted as the most prominent sensing platforms for 2022 revenue [15].

Although, other analytical techniques for ion detection, such as ion-chromatography (IC) or inductively coupled plasma (ICP), traditionally have lower limits of detection than potentiometry, those require the use of more complex and larger instrumentation that makes on-body portability impossible. Instead, these techniques are commonly the gold standard in the validation protocols of wearable sensors after sample collection [4]. Therefore, it is necessary to sacrifice a superior limit of detection during analysis to fulfil the portability requirement. Otherwise, a delay in obtaining the information relative to body status is generated from the total required time, which involves sample collection and transportation to a centralized laboratory and analysis time inherit to the technique. This leads to a postponement on the scale of hours or even days that is not desired in the decision-making process by the trainer or doctor (i.e., end-user in general). Nevertheless, it is here anticipated that the limits of detection displayed by potentiometric ISEs successfully cover ion levels generally presented in the different biological fluids and, therefore, this is not a true limitation for their application as wearable sensors [4]. On the other hand, in the case of a complex matrix, such as blood, possible limitations arise from selectivity issues (matrix effect) and (bio)fouling [16].

The interest in measuring ions in biological fluids rely on the relationship between the specific status of the individual as well as certain illnesses. For example, sodium and chloride levels in sweat are biomarkers used to assess hydration status in athletes, while elevated ammonium content is related to extreme fatigue [17]. In addition, pH monitoring helps in the treatment of chronic wounds, and other cations and anions are essential biomarkers in sweat to understand electrolyte imbalances within the body, which is associated with diverse dysfunctions in the human organism, as well as other diseases such as hypo- and hypernatremia, hypo- and hyperkalemia, heart disorders, muscle activity deterioration, disturbances in cerebral metabolism and cystic fibrosis, among others [18,19]. Among other examples, there is implying the detection of ionizable metabolic products related to different organism dysfunctions, as is the case in creatinine detection through its cation, creatininium, for the early identification of chronic kidney disease and other disorders [20] as well as charged drugs utilized in patient medical treatments that necessitate strict control of body levels, such as lithium-based formulation, trazodone and others [21,22]. Moreover, the incorporation of different sort of molecular recognition elements (such as aptamers and molecular imprinted polymers) permits the detection of both ionic and neutral biomarkers by means of potentiometric readout [23,24].

The very first prototype of a wearable potentiometric sensor was reported by Diamond et al. in 2010 and consisted of a sodium ISE of inner-filling solution type that was embedded in a belt with an absorbent patch for sweat collection [25]. Over the last eight years, the design of wearable potentiometric sensors has dramatically advanced, and this has been accompanied by the development of all-solid-state ISEs. As a result, a wide catalogue is currently available as recently reviewed by Crespo and co-workers [4]. Some valuable conclusions could be extracted from the critical analysis of the papers collected in such recent review by our group:

- There is a noticeable trend toward measuring ions in sweat (ca. 80% of the reported papers in the last eight years comprise sweat analyses) that is clearly owing to the high ion content in this matrix (on the mM range), the simplicity of the sample (with affordable selectivity requirements for ISEs) and the easy adaptation of daily objects/materials (such as sweatbands, epidermal patches and textiles), which are in contact with the skin for potentiometric measurements with ISEs.
- Despite sweat collection not involving any invasive and/or painful procedure in the individual, the secretion of a sufficient amount of sweat is needed. While this statement is compatible once the wearable sensor is applied to a sport activity, in the case of medical applications, it is necessary to expose the patient to high temperatures to increase the sweating rate, which will vary for different patients, or using alternatives techniques, such as iontophoresis-based instrumentation. The first option may sometimes disturb patient well-being, especially in elderly people, because of side problems related to exposure to high temperatures, such as low blood pressure or dizziness, whereas the iontophoresis-induction process may cause skin burning and skin allergies. Consequently, wearable potentiometric sensors have been mainly applied to sweat analysis during physical exertion, although the authors usually claimed that the devices could be used also for medical purposes [14,26,27].
- The all-solid-contact configuration is incorporated in all assessed wearable sensors contributions. In general, the devices are based on ISEs that were already characterized in a traditional configuration before its implementation into the wearable platform. The general strategy consists on the modification of a flexible substrate with a conductive path, then with the ion-to-electron transducer and finally with the sensing element (Figure 1c). Thus, ISEs comprising polymeric ISMs are used for the detection of ${\mathrm{Na}}^{+}$, $K^{+}$, ${\mathrm{Ca}}^{2+}$ and ${\mathrm{NH}}_{4}^{+}$, the conventional Ag/AgCl electrode for ${\mathrm{Cl}}^{-}$ sensing, while pH detection is conducted by solid electrodes based on polyaniline (PANI) or iridium oxide.
- As the selected papers were focused on wearables fitting sport activity monitoring, many authors investigated the resiliency of the sensor response facing different physical tests (such as stretching, bending, torsion, poking, crumpling and indention) as well as putting forward the use of novel electrode designs and advanced materials to circumvent any possible influence on electrode response [28].

As a consequence of the large imbalance between the high number of reported works regarding wearable potentiometric sensors applied to sport performance monitoring and the few papers dedicated to medical applications (e.g., a percentage of ca. 20% was shown in [4]), the present review analyses the most recent papers on wearable potentiometric sensors for medical applications, seeking to identify the causes for the bottleneck that has halted advancement in this field. For this purpose, it is first discussed which of the available biological fluids is compatible with the current wearable technology as well as with potentiometric sensing. Then, the review focuses on a critical description of wearable potentiometric sensors that have exhibited successful on-body application with the provision of clinically relevant information not connected to the physical status of the individual as a consequence of sport practice. In this sense, an extra number of wearable potentiometric sensors recently reported for the monitoring of sweat during physical performance was additionally analyzed because the proposed designs may be further applicable to purely medical purposes. The review aligns with our own proposal, mainly based on our experience in the field together with valuable insights reported in the literature, for the characterization of the analytical performance of wearable potentiometric sensor towards a fruitful prototype suitable for medical applications. It is also important to mention that the present work focuses on the sensing component of wearable potentiometric sensors and, therefore, the technical and connection requirements as well as data harvesting and transmission are outside of this scope. In this context, conformable and stretchable electronics able to wirelessly transmit data gathered from the potentiometric sensors, especially for sweat analysis, have been widely developed during the last 5 years [28,29].

## 2. Discussion on Potential Biological Fluids to Be Analyzed by Wearable Potentiometric Ion Sensors 

The detection of certain analytes in human blood has reliably supplied clinically relevant information for many years. Thus, medical diagnosis often depends on the retrieval of a blood sample that is later analyzed within a clinical laboratory. Despite this practice supplying consistent results, there is a delay in the gathering of data by the clinician as a consequence of the required sample transportation, manipulation and analysis time, along with the fact that blood extraction is an intrusive process involving physical trauma and often posing the risk of infection. These drawbacks have promoted the search for non-invasive devices that employ other biofluids, such as sweat, saliva and tears, that are also a source of relevant biomarkers [14], constituting this the basis of the conception of wearable sensors for healthcare applications.

Sweat is of particular interest because of its easy accessibility in terms of carrying out analytical measurements. This is one of the reasons why the vast majority of the reported wearable potentiometric ion sensors are proposed for sweat analyses [17,30]. In contrast, saliva and tear analysis comprise the implementation of the sensors in a more complex way. For example, Mannoor et al. demonstrated a dental tattoo for continuous and wireless monitoring of bacteria [31]. Kim et al. developed a wearable non-invasive mouthguard biosensor for continuous salivary detection of lactate and uric acid [32,33]. Sensors based on soft contact lens with integrated wireless electronics have been explored for monitoring other analytes, such as glucose and lactate, in tears [34]. Indeed, a device based on this concept is now being developed by Google and Novartis [35,36].

The possibility of the miniaturization of potentiometric cells is totally in line with this sort of design, although the compatibility of the materials used in the preparation of the indicator and reference electrodes with on-body implementation within the individual must be verified. This is especially important for the components of the membranes (sensing and reference ones).

Another alternative is the use of wearables for interstitial fluid analysis. Importantly, glucose sensors for diabetes have evolved from devices that evaluate capillary blood glucose levels typically by pricking the finger with a needle (late 20th century) to continuous glucose monitoring within the skin’s interstitial fluid [37].

The implementation of wearable potentiometric sensors to assess the urine of the patient is additionally feasible. One possibility here is to integrate sensors in a diaper or within a clinical bag in order to collect urine. In this direction, diapers featuring physical sensors that advise when the individual experiences a leakage in urine of 80 cc have been described in patients with incontinence [38].

Overall, none of these concepts for saliva, tears, interstitial fluid or urine analysis have been yet explored with wearable potentiometric ion sensors for medical applications as far as we know. In our opinion, this deficit is not a matter of physical and chemical adequacy of potentiometric sensors to this end, but it should be connected to the simplicity of sweat analysis and, therefore, this is currently used instead. Unfortunately, as other biofluids have not yet been investigated, the potential for using these samples together with wearable potentiometric sensors have not been yet assessed. All in all, scientists working in this field should be encouraged to achieve this purpose.

## 3. Description and Critical Evaluation of Recent Wearable Potentiometric Ion Sensors with Potential Medical Applications 

First, we reflect on certain interesting papers from the groups of Javey, Diamond and Wang that have been applied for sweat analysis during sport activities, but that could be employed for medical purposes in further investigations [26,27,39,40,41,42]. In our opinion, these papers comprise crucial advances in terms of the validation of on-body measurements and the design of the sampling cell important for discussion in the present review.

**Figure 2:**
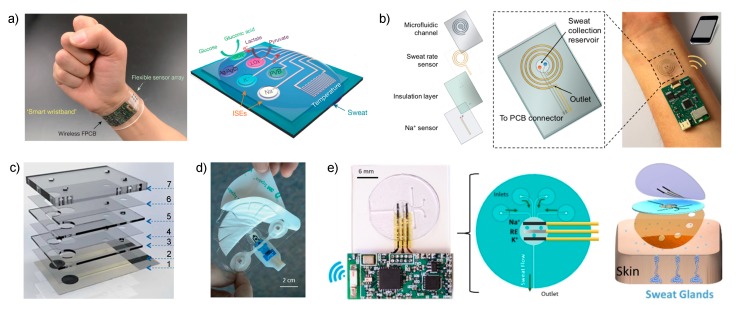
(**a**) Images of the wearable sensor reported by Gao et al. that is placed on a subject’s wrist, integrating the sensor array and wireless transmission. The array consisted of glucose-, lactate-, sodium- and potassium-selective electrodes together with the reference electrode (PVB reference membrane) and the temperature sensor. GOx and LOx are glucose and lactate oxidase, respectively. Reproduced from [26] with permission from Springer Nature. (**b**) Scheme of the four layers that constitute the wearable sensing patch reported by Nyein et al. The spiral Au electrode for sweat rate sensing is aligned with the microfluidic channel. The sodium and reference electrodes are placed in the sweat collection reservoir. The path can be worn on the user’s wrist and the data is wireless transmitted to a phone via Bluetooth. Reprinted from [39], with permission from the American Chemical Society, Copyright 2018. (**c**) Image of the different layers employed to produce the wearable sensor reported by Matzeu et al. Reproduced from [41] with permission from The Royal Society of Chemistry. (**d**) Image of the sweat patch developed by Alizadeh et al. The dimensions are 10.5 cm × 9.7 cm. Reproduced from [42] with permission from The Royal Society of Chemistry. (**e**) On the left: Photograph and schematic of the microfluidic device integrated with the ${\mathrm{Na}}^{+}$ and $K^{+}$ electrodes described by Sempionatto et al. On the right: Skin-mounted device, which comprises a three-layered configuration. Reproduced from [27] with permission from John Wiley & Sons, Inc.

Javey et al. reported in 2016 in *Nature* a sensor array for multiplexed *in situ* analysis of ${\mathrm{Na}}^{+}$ and $K^{+}$, but also glucose, lactate and temperature in sweat (Figure 2a) [26]. The sensor was applied to obtain a detailed sweat profile of healthy individuals subjected to a prolonged physical activity in indoor (stationary leg cycling) and outdoor conditions (running trials). The sensor provides a real-time assessment of the physiological status of the individuals for the first time, revealing critical information: (i) ${\mathrm{Na}}^{+}$ increases and $K^{+}$ decreases during the beginning of the perspiration, (ii) both stabilize as the activity continues, (iii) similar quantitative changes were noted in different parts of the body (forehead and wrist) but with different concentration levels, (iv) the electrodes also detect physiological responses of the subjects to a sudden change in exercise intensity (both with increasing effort and cooling down), (v) $K^{+}$ levels are always more stable than ${\mathrm{Na}}^{+}$ ones, which was attributed to its passive ion partitioning mechanism and (vi) dehydration trials featured a substantial increase in ${\mathrm{Na}}^{+}$ and a smaller raise in $K^{+}$ after 80 min of running without drinking water.

Potentiometric ISEs for ${\mathrm{Na}}^{+}$ and $K^{+}$ were based on ISMs using poly(3,4-ethylenedioxythiophene): polystyrene sulfonate (PEDOT: PSS) as ion-to-electron transducer, and the reference electrode was based on carbon nanotubes covered with a polyvinyl butyral (PVB) reference membrane. The paper presented the analytical characterization of the sensors during *ex situ* operation involving: the evaluation of the linear range of response (10–160 mM and 1–32 mM for ${\mathrm{Na}}^{+}$ and $K^{+}$, respectively); repeatability and long-term studies of sensitivity (i.e., ISEs slope) over a period of four weeks; selectivity for ${\mathrm{Na}}^{+}$, $K^{+}$, ${\mathrm{NH}}_{4}^{+}$, ${\mathrm{Mg}}^{2+}$ and ${\mathrm{Ca}}^{2+}$; and influence of mechanical deformation (bending) on sensitivity. However, the calibration graph of the ISEs were not studied before or just after each on-body usage to confirm the precision of the measurements and there was no quantification of the response time of the sensors apart from the control of perspiration time during on-body trials.

The accuracy of on-body measurements was qualitatively verified through visual comparison with the results obtained in *ex-situ* measurements using the same electrodes calibrated in artificial sweat. Sweat sampling was accomplished by scratching the forehead with microtubes. Unfortunately, there is a lack of quantitative information and the use of a gold standard technique for comparison during the validation protocol went unnoticed. Nevertheless, the more underscored points in this work are the provision and interpretation of real data, the technological design of the sensor as well as the data acquisition and visualization, the use of temperature measurements for sensitivity correction, the utility of one-point calibration before each employment of the sensor to correct any shift in the standard potential of the electrodes and the attempt to validate the real observations.

The same device was used later to detect ${\mathrm{Ca}}^{2+}$ and pH in sweat and urine [40]. The interest in this new configuration lies in the simultaneous detection of ${\mathrm{Ca}}^{2+}$ and pH because there is a clear correlation between them in several biological fluids, and such knowledge may aid in disease diagnosis, like in the cases of primary hyperparathyroidism and kidney stones. Notably, clinical correlations are essential for the double-check of daily diagnosis and, therefore, this sensor will fit within this aim. While the Ca–ISE was based on an ISM, the pH sensor consisted of a PANI film. The analytical performance of these two sensors were evaluated similarly as in the previous paper [26]. However, the validation protocol was slightly different. First, the electrodes were validated while *ex situ* functioning by means of collected sweat and urine and additional measurements using ICP and a commercial pH meter. During the on-body measurements in sweat–while the subject was training on a stationary bike–sweat was simultaneously collected for validation with ICP and a pH meter. The weakest point of this protocol is there is no statistical comparison to assess whether the observed accuracy lies within the tolerance limits for medical analysis (10–15% deviation in comparison with a gold standard) [43,44]. The authors only stated that similar trends and close readings are observed between the different techniques. Nevertheless, very interesting on-body observations were recorded: the pH of sweat was noted to rise gradually at the beginning of the activity, coinciding with a reduction in ${\mathrm{Ca}}^{2+}$ concentrations, and then both stabilized. The wearable was not applied for urine analysis beyond benchtop measurements.

In one of the most recent papers published by the Javey group, their research was focused on solving current challenges involving sweat rate monitoring in order to understand the complex relation between sweat composition and its sampling (dynamic studies) [39]. Consequently, the authors implemented a sweat collection reservoir into the design of the wearable rather than relying on the direct contact of the electrodes with the skin, as in the two preceding papers. While there was a clear trend of developing sweat sensors based on skin patch platforms 5 years ago [45,46,47,48,49,50], an evaluation of the recent literature points out that now the interest relies on the incorporation of a sampling cell aiming to overcome the drawbacks associated to accomplish the analytical measurements by means of sample accumulation on the electrode surface. Notably, this was previously discussed in our recent review [4].

Essentially, the new cell reported by Javey is based on a sweat collection reservoir, where ISEs for pH, ${\mathrm{Na}}^{+}$, $K^{+}$ and Cl^−^ are embedded (Figure 2b), connected to a spiral-patterned microfluidic component that permits progressive sweat flow governed by the pressure induced by naturally secreted sweat. Once the sweat fills up the collection reservoir, it is transported along the channel as a function of capillary action and the created pressure while fresh sweat continuously replaces the ‘old sweat’. Thus, the efforts are entirely concerned with the characterization of the new sampling cell by placing an impedance-based sweat rate sensor rather than analytical characterization of the sensors.

The authors outline a series of very interesting experiments designed to investigate the interplay between the measured concentration for ${\mathrm{Na}}^{+}$ and sweat rate, which was manually changed by a commercial pump (peristaltic). It is here anticipated that the main drawback of using sweat as healthcare diagnosis tool is the possible dependence of the measured ion concentration with the sweat rate. Advantageously, the sensor provides instant knowledge of the outward sweat rate and this permits correlating changes in ${\mathrm{Na}}^{+}$ measurements with changes in the flow rate or with pure changes in sweat composition. The sweat rate sensor was successfully validated theoretically and via optical measurements by a standard sweat collection system widely utilized in cystic fibrosis diagnosis. On-body measurements with this device were carried out for pH, $K^{+}$ and ${\mathrm{Cl}}^{-}$ detection in one individual subjected to indoor cycling, but: (i) the time needed to fill the sweat reservoir was not indicated; (ii) it is not clear if the sampling cell was protected from any evaporation and/or leaking issues and (iii) on-body observations were not validated. Importantly, data were recorded on a mobile phone through a customized app.

Along the same lines of implementing a sampling cell for sweat collection to afford reliable on-body potentiometric measurements, Diamond and co-workers recently reported two designs that are worth mentioning [41]. In the first (Figure 2c), the electrodes are embedded within a microfluidic cell composed of eight layers that generates a passive sweat pump. The purpose of each layer is as follows: (1) sensor paths; (2) isolation; (3) wholes for membrane deposition; (4) microchannel between the two electrodes; (5) absorbent material to cover the electrodes and fill the channel; (6) similar to (4); (7) closing of the cell containing a cotton thread to assist sweat movement into the microfluidic chip. The visualization is via a colored liquid showing that the fluid is able to travel along the system from the inlet to the outlet, but any other deeper characterization, including evaporation issues, is absent. The device was analytically tested for ${\mathrm{Na}}^{+}$ potentiometric sensing by means of electrodes comprising poly-3,4-ethylenedioxythiophene (PEDOT) as an ion-to-electron transducer and Na–ISM in terms of sensitivity (slightly sub-Nernstian slope, 53.1 ± 0.8 mV) and linear range (10^−3^ to 10^−1^ M, which seem to cover cystic fibrosis diagnosis in sweat).

Some on-body trials for ${\mathrm{Na}}^{+}$ detection during cycling sessions were accomplished, but despite the cotton threat could be employed to quantify average ${\mathrm{Na}}^{+}$ concentration in the collected sweat measured by a gold standard technique, no validation was reported. A three-point calibration was carried out before and after portability to evaluate any variation in the potential offset that may lead to errors in the quantitative calculation of ${\mathrm{Na}}^{+}$ concentration. Although the calibration recorded after the cycling session was selected for this purpose, it is not clearly stated or demonstrated for any reason why there was this selection. Regarding future clinical applications, the potentiometric microfluidic chip is beneficially connected to a wireless electronic platform to achieve a real wearable device applicable to detect ${\mathrm{Na}}^{+}$ as a biomarker. This may serve in the diagnosis and tracking of the progression of cystic fibrosis beyond enabling personalized hydration strategies for athletes or people under severe environmental conditions (such as firefighters and soldiers).

The second example consists of a wearable patch for continuous monitoring of ${\mathrm{Na}}^{+}$ and $K^{+}$ in sweat during exertion [42]. The paper concentrates on two crucial aspects of wearable fabrication: sample collection with flexible microfluidics and electronics components to afford a true portability of the device. Considering the microfluidics module, this was divided into three sections: the skin interface, sensor interface and wick. For the skin interface, the shape was optimized, trying to minimize the skin-to-sweat collector gap, therefore providing the least amount of time to fill the volume of the sensor flow cell (1.1 μL). This consisted of a segmented circle that uses a minimal adhesive area to maintain the gap constant while being of a disposable nature (Figure 2d). Then, this is in turn connected to a fluidic channel (made in a patterned adhesive layer) that transports the collected sweat through the sensor chip towards the wick on the downstream side. The wick also acts as the outlet of the fluidics up to a fan-shaped reservoir with a large area to accumulate the collected sweat and promote adequate flow (Figure 2d). All the system is mounted in a skin patch to be fix onto the skin for on-body measurements. The main experiments in this paper were focused on the characterization of the fluidics in the sampling cell by using a ${\mathrm{Na}}^{+}$ electrode operating under controlled flow rate conditions (1–20 μL·min^−1^ mimicking sweat rates). A calibration graph using the whole device and the response reversibility were fully demonstrated. Although the sensor displayed a response time on the range of several minutes with respect to controlled changes in NaCl concentration when the device is coupled to a peristaltic pump, it was asserted that temporal resolution of less than 10 min might be sufficient for the monitoring of hydration in individuals. Considering the medical application of the device, this time should also be enough for clinical analysis based on one-spot observation rather than continuous monitoring.

On-body testing of the sweat sensor was additionally carried out and, albeit any validation of the measurements was assessed (indeed, the authors stated that this is not the aim of the work presented), a series of valuable advancements were implemented. The drift of the signal recorded by the ${\mathrm{Na}}^{+}$ and $K^{+}$ electrodes was corrected considering a noise suppression algorithm (15-s moving-average approach) in order to consider the intrinsic electronic noise and connection to the patch itself with physical activity. On the other hand, infrared imaging of the temperature distribution in the subject during the exercise trial uncovered localized heating in the surroundings of the sweat patch, which the authors attributed to the insulating and blocking effects of placing external materials over the skin.

The last example selected for discussion in this section is the new skin-worn soft microfluidic potentiometric detection system reported by the group of Wang [27]. The device comprises a sampling cell made of polydimethylsiloxane (PDMS) silicone with four holes (2-mm diameter) acting as sweat inlets that are connected with microchannels to the detection chamber in which potentiometric sensors for ${\mathrm{Na}}^{+}$ and $K^{+}$ detection are embedded together with the reference electrode (Figure 2e). The sweat flow is transported from this chamber, requiring ca. 15 min to be completely filled, to an outlet channel that is placed parallel to the skin, thereby allowing for the continuous replenishment of sweat in the cell with minimized back pressure. Furthermore, during on-body experiments, the device outlet is placed facing upward to avoid any contribution from gravity forces in the sensing reservoir filling process. The device is mounted on the skin by attachment with medical grade adhesive with holes to provide the inlet and outlet of the sampling cell while respecting the natural sweat process of the sweat pores located within it. The properties of the flow in the devices were studied by computational simulation and then experimentally confirmed.

The electrodes for ${\mathrm{Na}}^{+}$ and $K^{+}$ detection were based on ISMs and a thin layer of polyurethane was cast on top, seemingly in order to prevent leaching. The analytical performance of the sensors once implemented in the device were analyzed in terms of calibration parameters, resilience (quantitative data not shown), carry-over effect (response reversibility to increasing and decreasing concentrations of the ion analyte), repeatability and stability (the device can be used several times during the same day and also over five days). Regarding interference studies, the authors commented that the use of artificial sweat background for the calibrations does not influence the calibration parameters (data not shown). On-body monitoring of ${\mathrm{Na}}^{+}$ and $K^{+}$ levels in sweat was carried out during indoor fitness cycling, with observed dynamic changes in the electrode response during prolonged exercise activity (40 min) but never reaching dehydration status of the individual. External calibration under flow conditions was used to calculate the concentrations from the recorded potentiometric signal. However, any possible modification of the calibration parameters after portability was not evaluated and therefore the accuracy of the quantitative observations could be questionable because validation is additionally missed. As a result, the reported data should be interpreted only qualitatively, as the authors executed in the paper.

Overall, as anticipated at the beginning of this section, there is a current trend of developing sampling cells based on new designs that enable trustworthy sweat collection while providing an effective fill and continued refill of sweat in the sensing zone where the electrodes are embedded. In this regard, it seems that the provision of a natural pump for the sweat flow through microfluidic channels, rather than implementing absorbent materials in the outlet, is more convenient. Furthermore, theoretical simulations help in both the design and later study of the flow in the developed system [39,42]. Thus, wearable devices based on this type of sampling cell (comprising inlet and outlet) have demonstrated certain success for the continuous monitoring of sweat while practicing a sport activity, despite there being plenty of room for establishing the appropriate validation protocol towards the assessment of the reliability of on-body measurements. It is worthwhile mentioning the impedance sensor recently developed by the group of Javey [39] in an attempt to supply real-time observations corrected by the response time inherent to the wearable potentiometric sensor operating as a whole entity.

Yet, while the incorporation of the sampling cell is mandatory to provide continuous monitoring of ions in sweat, this is not the case whether the wearable is applicable for medical purposes. Essentially, wearables implementing a sampling cell may also be applicable for this goal, as the authors generally stated in their works, but there are other possible designs based on the accomplishment of the sample collection in an accumulative way that are simpler and somehow more suitable for medical applications, as explained in the next section.

## 4. Description and Critical Evaluation of Wearable Potentiometric Ion Sensors Reported in the Last Five Years for Biomedical Applications 

Table 1 lists the most recent publications on wearable potentiometric sensors that have been demonstrated for medical applications, including, in some cases, real emulations and *ex vivo* experiments. A first inspection reveals that pH, ${\mathrm{Na}}^{+}$, $K^{+}$ and ${\mathrm{Cl}}^{-}$ was measured in wounds, sweat and interstitial fluid for medical applications.

**Figure 3:**
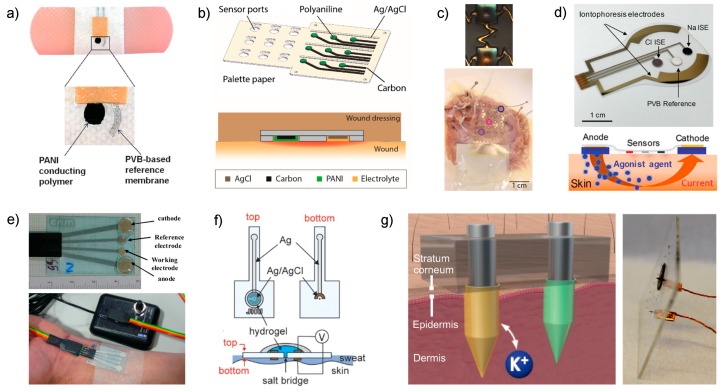
(**a**) Image displaying the potentiometric sensor printed on a band aid developed by Guinovart et al. Reproduced from [51] with permission from John Wiley & Sons, Inc. (**b**) Three-dimensional scheme of the pH sensor array by Rahimi et al. with a magnification of the cross-section of the area in which the sensors are embedded into a wound dressing. Reprinted from [52], with permission from Elsevier, Copyright 2018. (**c**) Wearable pH sensor reported by Chung et al. Top: Picture of one gold electrode after IrOx electroplating. Bottom: Human right ventricular wedge preparation with endocardial surface in contact with the wearable pH sensor array. Reproduced from [54], with permission from John Wiley & Sons, Inc. (**d**) Scheme of the wearable device by Emaminejad et al. based on iontophoresis for sweat extraction [57]. (**e**) Wearable sensor for ${\mathrm{Cl}}^{-}$ detection reported by Gonzalo-Ruiz et al. based on iontophoresis-induced sweat collection [58]. Reprinted from [58], with permission from Elsevier, Copyright 2018. (**f**) Schematic illustration of the sweat ${\mathrm{Cl}}^{-}$ sensor based on a salt bridge [59,60]. Reprinted from [59], with permission from Elsevier, Copyright 2018. (**g**) Illustration and picture of the microneedle-based patch for transdermal $K^{+}$ detection in interstitial fluid. Reprinted from [61] with permission from the American Chemical Society, Copyright 2019.

There are two papers that reported wearable pH sensors for the monitoring of wound healing [51,52]. In a first approach, Wang and co-workers followed a screen-printing fabrication technique for the working and reference electrodes on an adhesive band aid (Figure 3a) [51]. In a final step, PANI and the PVB-based reference membranes are integrated using a drop-casting method. In the case of PANI, the drop serves for *in situ* electrochemical polymerization of the polymer. The same approach (drop-casting) was employed to calibrate the electrode by removing the solution and gently clearing the electrode surface in the interim of each concentration change, i.e., the traditional ISE calibration based on separate solutions [53], but translated into a drop. As this is a discontinuous method, the plot showed dynamic calibration in the paper (we are referring, for instance, to Figure 2 in [51]), which is based on a continued trace of the potential, may be slightly confusing to the reader. Furthermore, the influence of evaporation on the measurements is not considered and it is not indicated how the spread of the drop over the electrodes is controlled considering that the part in which they are embedded consists of an absorbent (cotton-based) material within the band aid.

Analytical performance of the pH sensor was evaluated in terms of working range (Table 1), response time (20 s, fast enough to monitor pH changes in wounds that normally ranges from days to even weeks), interferences (${\mathrm{Na}}^{+}$, $K^{+}$, ${\mathrm{Cl}}^{-}$ and ${\mathrm{SO}}_{4}^{2-}$ do not interfere considering blood levels), hysteresis, reproducibility, repeatability, mechanical stress (bending), temperature (120 °C for 15 min) and lifetime (35 days). While the variation of the sensitivity (calibration slope) associated with possible facts was always <2%, except for temperature influence (4%), changes found for the intercept of the calibration graphs were more marked. A study based on the influence of these variations of the calibration parameters in the precision of the pH quantification would have indicated the final precision of the pH measurements.

One clear advantage of the reported device is that the electrodes do not need a pre-conditioning step or the full reversibility of the response. After assessing a deeper study of the analytical performance of the sensor in diluted human serum, the authors sought to emulate wound pH monitoring by means of covering the electrodes with poly(ethylene glycol) (PEG) hydrogel and varying the pH in this media by successive addition of diluted serum. Unfortunately, further studies involving the biocompatibility of the sensor showing any possible toxicity when in touch with the wound and through *in vitro* tests have not yet been accomplished to the best of our knowledge.

In this context, it has been demonstrated that the baseline pH of a number of analyzed wounds was greater than 8.5 [63]. Then, as the wound condition improves and exudate levels diminishes, the pH decreases to less than 8.0. Indeed, there is a proven association between the type of organism present and wound pH. On the other hand, if the pH of the wound does not evolve to lower values but to elevated values instead, this is a sign of infection as already confirmed for second-degree burns [64]. However, in the majority of the reported studies, pH paper strips were used to evaluate the wounds and further demonstration of the application of wearable pH sensors for this aim is absolutely necessary.

In another approach, Rahimi et al. developed a pH sensor array for wound assessment comprising a polymer-coated commercial paper (palette paper) modified with a PANI electrode and a Ag/AgCl reference electrode (Figure 3b) [52]. The paper contains a total number of nine pH electrodes in order to measure the pH in multiple wound regions simultaneously. Advantageously, materials and techniques selected for the fabrication of the sensors are compatible with low-cost, mass production and biocompatibility with human keratinocyte cells was additionally confirmed. Unlike the previous work, PANI film was here implemented by drop-casting and the authors claimed that an increase in the thickness of the film is likely responsible for a longer response time of the sensors (rise time of 12 s and fall time of 36 s), but still enough at the time scale of the wound evolution for detectable pH changes. Other analytical features, such as working range (Table 1), reversibility, slight increase of sensitivity with temperature (25–35 °C close to human skin), sensor stability and drift (during the first 5 h, no significant change but then the sensor displayed a drift of 0.5 mV/h that is equivalent to 0.01 pH/h, being non-significant for wound monitoring) along with material robustness were also assessed. However, the sensor performance was not demonstrated in real applications.

Continuing with pH monitoring, but this time with demonstrations of explanted rabbit and donated human hearts undergoing ischemia, Chung et al. reported a wearable containing interconnected arrays of miniaturized potentiometric pH sensors that are encapsulated in thin, low-modulus elastomers that would allow future non-invasive measurements on the surface of the beating heart [54]. The pH sensors were based on gold electrodes with electrochemically deposited IrOx (Figure 3c, upper part). The working range (Table 1), sensitivity, response time (0.5 s) and interferences ($K^{+}$ and ${\mathrm{Mg}}^{2+}$ as the most abundant cations in the extracellular space during ischemia) were evaluated with sensor arrays of 30 pH electrodes. Temperature influence was additionally assessed in a range of 20–60 °C. However, the study of other important features, such as reproducibility, repeatability, reversibility, long-term stability and lifetime, was not shown.

The aim of having electrode arrays (30 pH sensors) is to provide a pH mapping of the organ. The first *in vitro* test of this concept was carried out by means of a smartly designed experiment in which the diffusion of acid solution from a reservoir to a surrounding phosphate buffered saline (PBS) solution was monitored. After this test, the paper demonstrated several medical applications. Firstly, a balloon catheter pH sensor device was fabricated and used with a single *ex vivo* rabbit heart preparation, which underwent 30 min of global no-flow ischemia followed by reperfusion. The measured pH was stable at the beginning of the experiment, and it then decreased slowly over 30 min under ischemia and recuperated quickly up to stabilization upon reperfusion. All these variations were biologically justified. In addition, a skin-like stretchable membrane based on the pH arrays was also prepared (Figure 3c, bottom part) and tested during global non-ischemia on both *ex vivo* rabbit heart preparations (n = 3) and a single human right ventricle preparation. Unique spatiotemporal mappings of pH were provided for stationary conditions, ischemia-reperfusion as well as different reperfusion times owing to the use of the wearable pH sensor. The authors envisioned other applications of the developed device for biological research, such as surgical tools and long-term implants, although they do not propose any validation of the *ex vivo* observations.

In the context of monitoring ischemia-reperfusion events, Tahirbegi et al. reported on a sensor array of typical all-solid-state electrodes made of copper pins covered by ISMs for pH and $K^{+}$ sensing that can be introduced into the stomach or other sectors of the digestive tract by endoscopy [55]. While this is a medical tool, it could also not be considered a fully wearable sensor because of the intrusive nature of the endoscopy and necessary surgical access. Nevertheless, in our opinion, this work is important to be commented upon because of the successful demonstration of the *in vivo* clinical application in domestic pigs. Thus, extracellular pH and $K^{+}$ levels were detected by direct contact of the potentiometric (two for pH and two for $K^{+}$) and reference electrodes with the stomach tissue. Gastric ischemia was produced in two anesthetized pigs by occlusion of the arteries of the stomach. Both type of sensors displayed a clear change in the potentiometric signal after blocking the blood flow of the tissue: from pH 2.3 and 2.1 down to 1.7 and 1.3, from 8.9 and 8.7 mM up to ca. 16 mM for $K^{+}$. Thereafter, during the reperfusion episode, these values gradually recovered close to the initial ones.

However, the analytical characterization of the electrodes is limited to the establishment of the calibration graph and between-electrode reproducibility, and there is no validation for *in vivo* measurements and, therefore, the reliability of the observations could be questionable besides the successful demonstration of the electrode array operation. On the other hand, while the electrodes provide a continuous recording of the potential during intervention, pH and $K^{+}$ levels are shown every 50 min rather than a continuous profile without any explanation.

The thread-based pH sensors reported by Mostafalu et al. could be also considered non-wearable devices because of the way in which they are implemented for on-body measurements [56]. The authors showed several applications for a pH sensor incorporated within a conductive thread. First, the thread was passed through chicken skin to mimic further subcutaneous measurements. Biological fluids reached the sensing part of the thread (based on PANI) as a consequence of the thread-based microfluidic channel by a wicking process. Then, three different *in vivo* experiments were carried out: gastric pH measurements (the thread-based sensor was directly inserted into the stomachs of rats using oral gavage needles as guides), subcutaneous pH measurements by implanting the electrodes under the skin or by placing such electrodes under the skin using needles. The pH measured under the skin over 1 min displayed a value of 7.0 ± 0.1 and the gastric pH fluctuated from 1 to 3. Once more, the analytical characterization of the pH electrode is underestimated, and validation was not presented. Indeed, it is difficult to establish a validation protocol for this kind of measurement and possibly, *in vitro* accuracy assessments could be considered sufficient.

Emaminejad et al. developed a wearable device meant for cystic fibrosis diagnosis that combines ${\mathrm{Na}}^{+}$ and ${\mathrm{Cl}}^{-}$ electrodes together with a glucose sensor and system for sweat generation in patients based on iontophoresis [57]. Indeed, the current gold standard technique for screening cystic fibrosis, which takes a few hours to complete, is based on ${\mathrm{Cl}}^{-}$ detection in sweat after the sample is extracted by iontophoresis through chemical induction via pilocarpine [65]. The approach reported by Emaminejad incorporates an electrode array to modulate the iontophoresis process, apart from the sensing electrodes, in the same wearable patch (Figure 3d, upper part). Thus, a thin layer of agonist hydrogel, which contains a cholinergic sweat gland secretory-stimulating compound (e.g., pilocarpine), is placed between the sweat-induction electrodes and skin (Figure 3d, bottom part). Different patterns of sweat secretion can be achieved depending on the stimulating compound formulation, while the electrodes make the process programable. An extra protection circuit sets an upper limit on the iontophoresis current as a safety mechanism to avoid overheating and burning the skin. Thus, acetylcholine hydrogel displayed a high sweat-rate response (354 nL/min/cm^2^, over 1–5 min) with a short-lived effect, whereas pilocarpine and methacholine-based hydrogels ensure a long duration of secretion (>60 min). Importantly, later studies have shown the use of nicotinic cholinergic agonist, carbachol, to provide prolonged and localized sweat stimulation [66].

Regarding the potentiometric sensors, Ag/AgCl electrodes were implemented for ${\mathrm{Cl}}^{-}$ detection and an electrode based on ISM for ${\mathrm{Na}}^{+}$. Both sensors showed near-Nernstian behavior on the range of 10–160 mM for these ions. In addition, long-term detection of these ions was demonstrated over 6 h, although no drift was calculated, and repeatability studies showed nearly identical absolute potentials (intercept of the calibration graph) with a variation of <1% in the sensitivity (slope). While the analytical characterization of the sensors is not overwhelming, the authors focused the studies reported in the paper on the in-depth characterization of the iontophoresis features of the wearable patch and demonstrated the diagnostic capability of the platform in the context of cystic fibrosis. In this sense, a sweat ${\mathrm{Cl}}^{-}$ level of ≥60 mM indicated a high likelihood of having cystic fibrosis and for concentrations <30 mM, the disease is unlikely [65]. Furthermore, it has been indicated that the ratio of ${\mathrm{Na}}^{+}$ over ${\mathrm{Cl}}^{-}$ concentrations in sweat may improve the success of the decision-making process based on real-time measurements [67], and therefore, this is the reason for the simultaneous implementation of the ${\mathrm{Na}}^{+}$ and ${\mathrm{Cl}}^{-}$ potentiometric sensors in the same wearable.

For on-body measurements, a water absorbent rayon pad was placed between the skin and sensor array close to the zone of sweat generation. The pad is able to absorb and maintain all the generated sweat while preventing direct mechanical contact between the sensors and skin. In principle, the incorporation of the pad does not disturb electrode performance, but there is no evidence of this fact in the paper. To stimulate the subject sweating, a 1 mA current is applied onto the skin by means of iontophoresis electrodes. This allows for the effective delivery of cholinergic agonists to the dermal space to reach the sweat glands and induce sweating (Figure 3d, upper part). After 20 min, the collected sweat is sufficient to close the electrochemical cell and, therefore, the sensors provide a constant potential that is recorded during the next 5 min from which the average ${\mathrm{Na}}^{+}$ and ${\mathrm{Cl}}^{-}$ concentrations are calculated. This approach was used to examine sweat content in six healthy volunteers and three cystic fibrosis patients, and the results clearly showed concentrations lower than 20 mM and higher than 60 mM for ${\mathrm{Na}}^{+}$ and ${\mathrm{Cl}}^{-}$ in healthy and diseased individuals, respectively. Despite this study being very interesting, no validation of the on-body observations was presented. Indeed, appropriate evaluation of the response drift is essential for reliable calculation of the ion analyte concentration when using this type of sensor in which concentrations are calculated using the average of the recorded potential over a certain period of time. Notably, this could affect medical decisions involving early diagnosis of any disease, and in such cases where biomarkers levels are close to limit situations.

In this latter sense, Choi et al. developed a sweat ${\mathrm{Cl}}^{-}$-wearable sensor (also based on iontophoresis sweat induction) consisting of a Ag/AgCl electrode with an integrated salt bridge that minimizes equilibration and enables stable measurements over extended periods of time versus the absence of a salt bridge [60]. As a result, the sensor displayed a very small concentration drift even at lower concentrations: a variation of less than 2 mM for 10 mM ${\mathrm{Cl}}^{-}$ concentration and 5 mM at 150 mM ${\mathrm{Cl}}^{-}$ concentrations. Still, the uncertainty related to the lower ${\mathrm{Cl}}^{-}$ concentrations is higher than that within the clinical tolerance (20% *versus* 10%), but this fact does not really compromise the decision-making process because the highest ${\mathrm{Cl}}^{-}$ concentration to assure that the individual likely has cystic fibrosis is 60 mM. However, the sensor was not analytically characterized in depth apart from the drift evaluation and biocompatibility studies involving the Ag/AgCl electrode as it is in contact with the skin.

The same group has recently presented the validation of this device by comparing the results observed with the wearable sensor *versus* the standard laboratory test for cystic fibrosis [59]. For this purpose, ${\mathrm{Cl}}^{-}$ sweat content in 10 individuals with cystic fibrosis and another 10 healthy individuals was on-bodily analyzed. A Macroduct sweat collection device was attached to one arm and the sweat collected for 30 min was then assessed in a chemical laboratory by coulometric titration (ChloroCheck® Chloride meter, Wescor). The wearable sensor was attached to the other arm and the ${\mathrm{Cl}}^{-}$ concentration was monitored in real time for 30 min using a Bluetooth transceiver and smart phone app. The ${\mathrm{Cl}}^{-}$ concentrations measured by the two methods exhibited robust agreement (Pearson correlation coefficient p = 0.97), displaying values <29 mM for healthy subjects and >60 mM for patients with already diagnosed cystic fibrosis.

In the same context of cystic fibrosis diagnosis, and previous to the paper by Emaminejad [57], Gonzalo-Ruiz et al. presented a wearable potentiometric sensor for ${\mathrm{Cl}}^{-}$ detection in sweat by pilocarpine-induced iontophoresis sweat collection [58]. While this work can be considered one of the pioneers in the field, the design developed at that time may be considered obsolete today (Figure 3e). Besides this, the analytical characterization of the device is rather incomplete. However, the authors accomplished the validation of on-body measurements in six patients by comparison with the method generally used in hospitals (sweat Chloride Analyzer from Advanced instruments Inc., Norwood, MA, USA), obtaining deviations lower than 8%.

Last but not least, microneedle-based potentiometric sensors have been recently published for transdermal detection of $K^{+}$ in interstitial fluid [61]. Previous devices have utilized microneedles either for the extraction of the interstitial fluid coupled to a fluidic system to transport the sample to the detector or simply for a simple collection followed by centralized analysis [61,68]. In particular, the microneedle-based extraction was coupled to potentiometric detection by means of a microchip containing a miniaturized $K^{+}$ sensor based on an ISM together with the reference electrode, allowing for the analyses of very low sample volume (the needed volume is not mentioned in the paper but should be on the order of very few μL) [68].

One step further, the group of Crespo have recently demonstrated $K^{+}$ detection in interstitial fluid by integrating the selective sensor as well as the reference electrode directly in the microneedle (Figure 3f) [61]. After an exhaustive analytical characterization of the electrode response (calibration parameters, response time, limit of detection, selectivity, repeatability, reproducibility, long-term drift, light/dark sensitivity, insertion effect) and the physical definition of the modified microneedles by scanning electrochemical microscopy (shape, length, tip angle, thickness in the tip and the base), the electrodes demonstrated features suitable for transdermal $K^{+}$ detection. Consequently, the patch was tested for *ex vivo* experiments via different kinds of animal skins (i.e., chicken and pork). Additional cytotoxicity studies revealed that the leaching of membrane components may compromise the use of the patch after wearing it for more than 24 h. Moreover, despite the microneedle patch being a wearable sensor for transdermal $K^{+}$ detection, its utilization implies a higher level of invasion in the subject compared to traditional wearables for sweat analysis. Hence, on-body measurements are not straightforward and other essential experiments, such as risk evaluation of membrane detachment while portability and related effect on the individual integrity, must be properly assessed before reaching the stage of *in vivo* experiments. On the other hand, further validation of on-body measurements is not as accessible for sweat analysis as the author reflected on throughout the paper. However, this inquiry constitutes a valuable contribution towards the early diagnosis of diseases related to electrolyte imbalance that is especially manifested in interstitial fluid.

The only example that we could find in the literature involving a wearable potentiometric sensor to analyze a biological fluid different than sweat and interstitial fluid is based on an intraoral device implemented in a porous dental retainer capable of analyzing ${\mathrm{Na}}^{+}$ in saliva (Table 1) [62]. This work by Lee et al. focused on the development of a low-profile, hybrid electronic system with integrated circuits and stretchable interconnects, which is embedded in an elastomeric platform, to provide a gentle, conformal integration in the oral cavity while offering a long-range wireless and real-time quantification of ${\mathrm{Na}}^{+}$ intake. The ${\mathrm{Na}}^{+}$ sensor is based on a traditional selective membrane and the reference electrode is an Ag/AgCl one. In vivo experiments reported in this paper comprised ${\mathrm{Na}}^{+}$ detection when a subject drinks water with different NaCl concentration. As the sensor is able to detect changes in ${\mathrm{Na}}^{+}$ level in the different situations, the authors claimed that the device could be used for hypertension management in a future. However, to the best of our knowledge, this type of clinical management is normally accomplished through the salivary potassium-sodium ratio. Consequently, the authors should incorporate a potassium sensor in the current design of the device, as well as accomplishing an initial validation of the in vivo observations and crucial toxicity and compatibility experiments before further steps. Nevertheless, this work constitutes an inspiring wearable potentiometric sensor for saliva analysis beyond the traditional sweat sensors.

After this deep analysis of the selected recent contributions in the field of wearable potentiometric sensors for medical applications, it is worthwhile mentioning that only sweat detection of ${\mathrm{Cl}}^{-}$ and ${\mathrm{Na}}^{+}$ in the context of cystic fibrosis has been successfully assessed over the last five years. Although the presence of ions in biological fluids has involved collection through non-invasive and painless approaches, such as saliva, tears and urine, current research is primarily concerned with sweat analysis. As anion detection is in general much difficult than cation detection due to the absence of appropriate selective ionophores [69], the lack of wearable potentiometric ion sensors to analyze other important anions (such as lactate) present in biological fluids is in principle expected. On the other hand, it is surprising that only the detection of ${\mathrm{Na}}^{+}$ has been demonstrated in a real scenario. In our opinion, the reason for this fact relies more in general difficulties related with the execution and validation of real measurements rather than with the potentiometry concept *per se*.

In addition, iontophoresis-induction for sweat collection seems to offer a successful approach to wearable potentiometric sensors for medical applications, rather than incorporating a sampling cell. Indeed, other wearable sensors have employed commercial iontophoresis units for sweat collection that may be further incorporated with potentiometric sensors [70]. As an example, ActiveDose II (ActivaTek, Gilroy, CA, USA) and the Wescor Nanoduct system merit mentioning [71]. Furthermore, one recent work presented by the group of Heikenfeld investigates in detail the sweat rate provided by a carbachol-based iontophoresis system using an impedance electrode similar to that reported by the group of Javey [39]. The device was additionally coupled to ${\mathrm{Na}}^{+}$ and ${\mathrm{Cl}}^{-}$ potentiometric sensors and its response was evaluated under three different conditions: no iontophoresis and no pressure applied, no iontophoresis with pressure and iontophoresis with pressure applied [72]. The authors demonstrated that the sensors can potentially be utilized to predict the sweat generation rate and therefore the sampling rate for an iontophoresis-based device, but the presented approach could be considered a bit complex. Overall, the development of iontophoresis-based devices aiming at sweat characterization for medical applications is in continuous boom.

## 5. Tentative Guidance for the Successful Characterization of a Wearable Potentiometric Ion Sensor

As a general trend when developing a wearable potentiometric sensor, the analytical characterization of electrode performance is underestimated because it has been reported for regular electrodes prior to implementation into the wearable configuration. Thus, calibration curves of the electrodes outside the wearable device are always presented but other features that are important for the reliable quantification of the ion analyte concentration typically go unnoticed. Thereafter, it is essential to establish a validation protocol that allows for accuracy assessment.

In this sense, we discuss in the following a number of issues related to the evaluation of the analytical performance of wearable potentiometric sensors as well as some guidelines that may help in further successful application to gather trustworthy medical information.

### 5.1. Calibration of the Sensors

The calibration curve selected to transform the potential readout of the wearable potentiometric sensors into the concentration of the ion analyte is crucial to obtain accurate results. There is a common practice of recording the calibration graph within the linear range of response just before the implementation of the electrodes into the wearable and then use the fitting (we will call this fit as initial calibration curve from this point forward) to calculate the concentration of the ion analyte in sweat. In general, the curve is based on three concentration points. However, this strategy has some issues that need to be clarified before accepting it as a universal approach for the calibration of the wearable. It is necessary to demonstrate that the recorded calibration does not vary owing to several reasons: (i) the implementation of the sampling approach, (ii) portability during on-body measurements and (iii) drift of the electrode response because of (bio)fouling or component leaching. As a consequence, a comparison of the calibration graph obtained before and after the incorporation of the sampling cell or absorbent pad, as well as before and after on-body measurements, is essential to confirm that the initial calibration graph can be used to calculate the concentration of the ion analyte in sweat in a reliable manner. Interestingly, resiliency tests based on the evaluation of the calibration graph before and after the electrodes face mimicked corporal movements (i.e., stretching, bending and twisting, among others), potentially reinforcing the right-directed use of the initial calibration graph.

Concerning electrode drift, it is necessary there is an evaluation of this parameter in a medium-term experiment to establish if any correction of the calibration parameters is required. As on-body experiments with wearable potentiometric sensors for medical applications usually involve a period no longer than 1 h, we would suggest assessing the electrode drift (also for the reference electrode) in artificial or real human sweat (or the biological fluid in question) over ca. 2 h for reliable assessment. In addition, the correction of the calibration slope with the temperature will offer a more reliable calculation of the concentration of the ion analyte. Indeed, a combination of both drift and temperature corrections have been already used for in situ environmental water measurements via potentiometric sensors [73,74].

Other matters surrounding the calibration graph is the selection of the background electrolyte and the use of either activity or concentration of the ion analyte. The use of artificial sweat (or other fluids) as background for the calibration graph is advantageous in that it allows the ionic strength to be known and, therefore, the interconversion concentration-activity can be easily determined through the two-parameter Debye−Hückel approximation [75]. Nonetheless, the majority of authors opt to use the concentration of the ion analyte directly in the calibration graph, assuming the activity coefficient is equal to one. Although this simplification seems to be generally accepted in the field, this has to be considered as a source of error in the quantification of the ion analyte as ion concentration in sweat is relatively high (i.e., from moderate to high ionic strength medium) and this approximation is only valid for very diluted solutions. A comparison between the results obtained following both approaches (i.e., using concentration versus activity in the calibration graph) should be accomplished to evaluate and therefore be able to discard any error in this practice. This could be accomplished measuring a sweat sample, which was already characterized by IC or ICP, with the wearable and then calculating the ion analyte concentration using both calibration graphs (using either concentration or activity). The statistical comparison of the calculated concentrations with those observed with IC or ICP will inform which approach is more reliable.

Whether all these aspects are considered in the elaboration of the calibration graph, quantitative results provided by a wearable potentiometric sensor will be more trustworthy, which is important for related clinical decision-making. Otherwise, the observed data may be interpreted only qualitatively, i.e., to identify critical cases with a probability of presenting a certain disease.

### 5.2. Other Analytical Parameters

Other analytical characteristics that are important to be assessed (mainly considering sweat as the sample) are:

- Reversibility. The so-called ‘carry-over test’ evaluates the possibility of measuring increasing and decreasing steps in the ion analyte concentration [27]. It is crucial to carry out this test once the electrodes are implemented with the sampling strategy.
- Response time inherent to the electrode and response time associated to the device involving the sample collection. When a sampling cell is used for this purpose, this latter comprises the time needed to fill the sensor compartment and depends on the sweat rate of the subject. It is here also critical to adopt a correction of the measurements depending on the sweat rate at each moment, just as in the case of the Javey group [39]. Notably, it would be convenient to more deeply analyze the behavior of the electrodes in this type of flow cell. We are referring to investigating the effect of convection, diffusion and the magnitude of the flow rate during the potentiometric response. In this regard, it is crucial to consider that the use of the initial calibration will be conditioned by the fact that these factors may slightly affect the electrode response, therefore introducing a source of error.
  On the other hand, it is hard to assure on-body monitoring in real time because a certain amount of sample is always needed to fill the reservoir in which the sensors are embedded. Thereafter, the reservoir continuously renews the sweat, but this does not occur exactly at the same time the individual is sweating. Time is needed for the generated sweat to reach the sensors’ chamber and then each sensor (in the case of multiplexed analysis), which involves a delay between the sweating process and data observation.
  In the case of an absorbent material coupled to iontophoresis-based sweat collection, the response time depends on the filling of the pad together with several minutes of potential recording [57].
- Between-electrode reproducibility and response repeatability (same electrode). These two features are vital for the final use of the wearable potentiometric sensors reaching mass production and commercialization. In the ideal case, a universal calibration graph valid for each ion analyte is implemented in software that permits the end-user to read the corresponding concentration in sweat. However, this is only valid when exactly the same calibration is observed for different electrodes fabricated in the same manner and for the same electrode over time. Yet, this does not happen in reality. Conversely, current efforts in the development of new ISEs focus on the direction of achieving this purpose, or what are called calibration-free sensors. Several strategies have been published over the last five years, such as the use of redox pairs as ion-to-electron transducers [76], the adjustment of the E^0^ of the calibration graph by applying a controlled potential or current as well as short-circuiting [77,78] and charge counting either by interrogating ISMs using dynamic electrochemical techniques rather than potentiometry or by the confinement of the sample to a thin-layer gap [79]. Regrettably, none of them has yet been implemented into a wearable configuration, as far as we know, and maybe, the next generation of calibration-free wearable ion sensors relies on this advancement.
- A characterization of the reference electrode is missing in most of the wearable potentiometric sensors. Indeed, a misbehavior of the reference electrode is a strong source of error in the calculation of the ion analyte concentration if, for any reason, the electrode does not provide constant potential. In this regard, it is important to characterize the reference electrode independently and then together with the potentiometric electrodes once implemented in the wearable. In a first step, the potential of the reference electrode must be tested for changes in electrolyte concentrations, redox species, pH, temperature, physical deformation as well as light/darkness conditions. Then, medium-term stability in (artificial) sweat (or other fluids) should be also assessed.

### 5.3. On-Body Measurements and Validation

After the proper evaluation of the analytical performance of the wearable potentiometric sensor, on-body implementation is not straightforward. First, all trials should be ethically accepted by the corresponding commissions and the subjects must sign official agreements. Then, it is necessary that all the materials used in the fabrication of the device are compatible with individual well-being in terms of allergies and toxicity, among others. In particular, considering devices based on iontophoresis for sweat induction (Table 1), it is important to evaluate any possible side effect in the patient coming from the use of the required chemicals and the applied current to avoid overheating and burning of the skin.

To guarantee trustworthy ion analyte concentrations, the appropriate validation of on-body measurements is mandatory. However, this is not an easy practice and consequently, none of the reported devices to date is fully validated. When sweat collection is stimulated either by sport practice or exposure to high temperatures, as the rest of the individual’s body also sweats, absorbent patches (or other systems for sweat collection) placed around the wearable may serve to then analyze the collected sweat with a gold standard technique, such as IC, ICP or via pH meter. Nevertheless, the provided data is not of a continuous nature, as in the case of the wearable because a certain amount of time is necessary to be able to collect enough sample volume (depending on the sweat rate, 5–10 min for 300–700 µL of sweat). In addition, either IC or ICP are not able to provide constant monitoring as potentiometric (or electrochemical) sensors. Hence, only a qualitative validation is purely possible by comparing the average concentration measured with the wearable *versus* that obtained using the IC or ICP. Notably, the sweat sample likely needs to be diluted in order to be analyzed within the IC or ICP, constituting another source of error added to the contamination risk involved in whole-sample collection and handling. Besides this, whether a proper validation is possible, on-body observations must be only considered as a medical alarm but never be the only parameter for final decision-making by the clinician.

Whether the wearable is based on an iontophoresis sweat stimulation, the only means to collect sweat for external analysis is by an absorbent material (or pad) placed in the device in such a way to permit the accumulation of the locally generated sweat on it, as the design reported by the group of Javey (Figure 3d) [57]. Thus, once on-body measurements are carried out, the same plug of sweat can be analyzed by IC or ICP after the pad is disassembled from the wearable in a safe fashion. Here, a quantitative comparison with the results obtained by the gold standard technique is possible and therefore the accuracy of the wearable can be fully assessed. Notably, for clinical measurements, an accuracy of 10% is generally the tolerance limit for a newly established technique [4]. Another option that may come to mind is integrating the iontophoresis-based sweat stimulation in a fluidic system with a collector system coupled to the outlet. Yet, to the best of our knowledge, this design has not yet been proposed in the literature.

Considering an ideal case, first on-body trials should involve a system in which more than one electrode of the same type are included in the wearable in order to obtain reproducibility of the observations. Furthermore, it may be also interesting to incorporate a second reference electrode to confirm that the selected design provides a constant potential during on-body tests. These kinds of experiments will reinforce the veracity of the analytical measurements and, consequently, further decision-making by the physician will be more reliable.

## 6. Conclusions

Wearable device technology combined with potentiometric ion sensors adhering to an all-solid-state concept has demonstrable potential in the monitoring of physical status during sport performance as well as clinical medicine through sweat analysis. Current investigations involving physical activity focus on the development of a sampling cell that supplies real-time analysis of the sweat of the individual. In contrast, devices that have demonstrated successful gathering of medical information are based on iontophoresis-based sweat induction that allows obtaining one-spot ion analyte concentration as the average of the measurements over the portability of the sensor. As far as we know, wearable potentiometric sensors have only been applied for the diagnosis of cystic fibrosis via on-body measurements of ${\mathrm{Na}}^{+}$ and/or ${\mathrm{Cl}}^{-}$. On the other hand, other devices have been tested exclusively with *ex vivo* tests. We are referring for example to transdermal detection of $K^{+}$ using microneedle-based sensors and pH mapping in the heart during ischemia-reperfusion episodes. It is expected that wearable potentiometric ion sensors gradually will be able to engage in the analysis of other forms of biological fluid that may provide valuable clinical information, such as saliva, tears and urine. Indeed, the bottleneck that slows down this purpose is not related with the potentiometry concept *per se*, as identified in the paper.

In general, we have found a lack of rigorous assessment of the analytical performance of wearable potentiometric ion sensors, yet this is extremely important to assure minimum error in the final calculation of the concentration of the ion analyte. This is especially crucial when selecting the calibration protocol of the sensors. In addition, appropriate validation of on-body measurements has generally not been accomplished. With the present review, we are seeking to establish a tentative guidance for the successful characterization and validation of a wearable potentiometric ion sensor, or at least generate debate within the scientific community that allows for the establishment of a well-conceived analytical protocol. The immediate future of wearable potentiometric sensors should rely on the provision of validated and therefore trustable data, before opening the range of clinical targets.

## Figures and Tables

**Table 1:** Description of recently reported wearable potentiometric sensors that have been employed for medical applications.

Analyte	Platform	Sensor	Working Range	Application	Medical Information	Sample Collection	Real Tests	Ref.
pH	Adhesive band aid	PANI	5–8	Wound	Wound healing ^a^	No	No ^b^	[51]
pH	Polymer-coated paper	PANI	4–10	Wound	Wound assessment ^a^	No	No	[52]
pH	Elastomer	IrOx	4–10	Heart	Ischemia-reperfusion	Explanted heart	Rabbit and human heart ^c^	[54]
pH $K^{+}$	Beryllium copper alloy pins ^d^	pH-SM KSM	0.7–1.5 0.1–10 mM	Gastric mucosa	Ischemia-reperfusion	No	Pig stomach	[55]
pH	Conductive threads	PANI	3.5–8	Subcutaneous and gastric	Sutures and implants ^a^	Wicking	Rats	[56]
${\mathrm{Na}}^{+}$ Cl^−^	PET	NaSM Ag/AgCl	10–160 mM	Sweat	CF	Iontophoresis + pad	Healthy and CF patients	[57]
Cl^−^	Polyester	Ag/AgCl	10–100 mM	Sweat	CF	Iontophoresis	CF patients	[58]
Cl^−^	PET	Ag/AgCl + bridge	10–100 mM	Sweat	CF	Iontophoresis	Healthy and CF patients	[59,60]
$K^{+}$	MN (Steel)	KSM	6·10^−5^–8·10^−2^ M	Interstitial fluid	K imbalance ^a^	No ^e^	Chicken skin ^c^	[61]
${\mathrm{Na}}^{+}$	Dental retainer	NaSM	1·10^−3^–1 M	Saliva	Hypertension management ^a^	No	Drinking saline water	[62]

^a^ Not demonstrated in the paper but planned for further experiments. ^b^ Emulation of wound monitoring by controlled pH changes in poly(ethylene glycol) hydrogel. ^c^ *Ex vivo* experiments. ^d^ Diameter of 600 µm. ^e^ Transdermal detection using MN as a sensor. PET=polyethylene terephthalate; pH-SM=pH selective membrane; NaSM=sodium selective membrane; KSM=potassium selective membrane; MN=microneedle; CF=cystic fibrosis.
